# Association between serum potassium and Parkinson’s disease in the US (NHANES 2005–2020)

**DOI:** 10.3389/fnins.2024.1387266

**Published:** 2024-05-09

**Authors:** Xue Zhou, Jingtong Zhao, Yang Liu, Xiaozhou Sun, Xuefeng Li, Jixiang Ren, Qingjie Li, Dong Han, Ting Pan, Yingqi Shi, Dalong Wu, Xinhua Chen

**Affiliations:** ^1^Changchun University of Chinese Medicine, Changchun, China; ^2^Changchun Central Hospital, Changchun, China; ^3^Center of Children's Clinic, The Affiliated Hospital to Changchun University of Chinese Medicine, Changchun, China; ^4^The Affiliated Hospital to Changchun University of Chinese Medicine, Changchun, China

**Keywords:** serum potassium, Parkinson’s disease, NHANES, cross-sectional study, neurodegeneration

## Abstract

**Background:**

Evaluating the correlation between serum potassium and Parkinson’s disease (PD) in US adults.

**Methods:**

A cross-sectional study was conducted on 20,495 adults aged 40 years or older using NHANES data from 2005 to 2020. The study utilized one-way logistic regression and multifactorial logistic regression to examine the correlation between serum potassium levels and PD. Additionally, a smoothed curve fitting approach was employed to assess the concentration-response relationship between serum potassium and PD. Stratified analyses were carried out to investigate potential interactions between serum potassium levels and PD with variables such as age, sex, race, marital status, education, BMI, smoking and medical conditions like coronary, stroke, diabetes, hypertension, and hypercholesterolemia.

**Results:**

In this study, a total of 20,495 participants, comprising 403 PD and 20,092 non-PD individuals, were included. After adjusted for covariates, multivariable logistic regression revealed that high serum potassium level was an independent risk factor for PD (OR:1.86, 95% CI:1.45 ~ 2.39, *p* < 0.01).The linear association between serum potassium and PD was described using fitted smoothing curves. Age, sex, race, education, marital, BMI, coronary, stroke, diabetes, hypertension and hypercholesterolemia were not significantly correlated with this positive connection, according to subgroup analysis and interaction testing (P for interaction >0.05).

**Conclusion:**

Serum potassium levels are elevated in patients with Parkinson's disease compared to non-PD patients. Additional prospective studies are required to explore the significance of serum potassium levels in individuals with Parkinson's disease.

## 1 Introduction

Parkinson’s disease (PD) is a neurodegenerative disease caused by extrapyramidal dysfunction, second only to Alzheimer’s disease in terms of morbidity, with chronic progressive aggravation, seriously affecting the physical and mental health and quality of life of the elderly. Its main pathological features are the aggregation of Lewy bodies in the substantia nigra and striatum and the loss of dopaminergic neurons. Studies have shown that the loss of more than 50% of dopaminergic neurons in the substantia nigra striata circuit leads to typical motor symptoms, including bradykinesia, resting tremor, myotonia, and postural disturbances (Jankovic and Tan, 2020).

Metal ions play a crucial role in maintaining health and disease, serving as essential cofactors for enzymatic reactions and contributing to neurophysiological homeostasis (Lothian et al., 2013). Imbalances in ion concentrations can disrupt homeostasis, resulting in cellular dysfunction and impacting overall organismal health (Fraga, 2005). Emerging research indicates that dysregulation of metal homeostasis is implicated in various neurodegenerative diseases such as Alzheimer’s disease, PD, vascular dementia, and amyotrophic lateral sclerosis. For instance, patients with vascular dementia often exhibit elevated levels of metal ions (Philbert et al., 2022).The concentrations of metals in the brain typically exceed those found in other bodily tissues (Roberts et al., 2012), and disruptions in metal homeostasis within the brain can result in heightened levels of brain lesions, oxidative stress, and inflammatory responses. As individuals age, metal accumulation in the brain, coupled with any disturbances in metal homeostasis, can contribute to neuronal damage, cell death, oxidative stress, and potentially protein misfolding and aggregation (Waldron et al., 2009).

Metals have long been recognized as playing a role in the pathophysiology of PD (Lothian et al., 2013). Elevated levels of certain metal ions have been detected in α-synuclein (α-syn) within neurons of PD patients (Davies et al., 2011; Camponeschi et al., 2013; Moons et al., 2020). α-syn is an intrinsically disordered protein that self-aggregates and significantly contributes to PD. Researchers have observed that altered potassium levels in PD may facilitate the accumulation of α-syn, contribute to the formation of PD-related Lewy bodies, and increase the risk of developing PD (Follett et al., 2013). Despite this, there is limited research on the relationship between potassium and PD. Potassium is a crucial electrolyte in the body, essential for nerve and muscle function. Imbalances in potassium, either hyperkalemia or hypokalemia, can lead to movement disorders with muscle weakness as the primary clinical symptom (Riggs, 1989). Several studies have indicated that PD patients may exhibit lower serum potassium levels (Rajput et al., 2021). This decrease in potassium levels could potentially contribute to muscle fatigue and dysfunction, thereby exacerbating dyskinesia in PD patients. Conversely, research has also suggested that elevated potassium levels, or hyperkalemia, could result in cardiovascular issues like arrhythmias in individuals with PD (Sridharan and Flowers, 1986). As such, this study delves into the relationship between serum potassium concentration and PD, primarily drawing from data in the NHANES database.

## 2 Materials and methods

### 2.1 Database and survey populations

**Figure 1:**
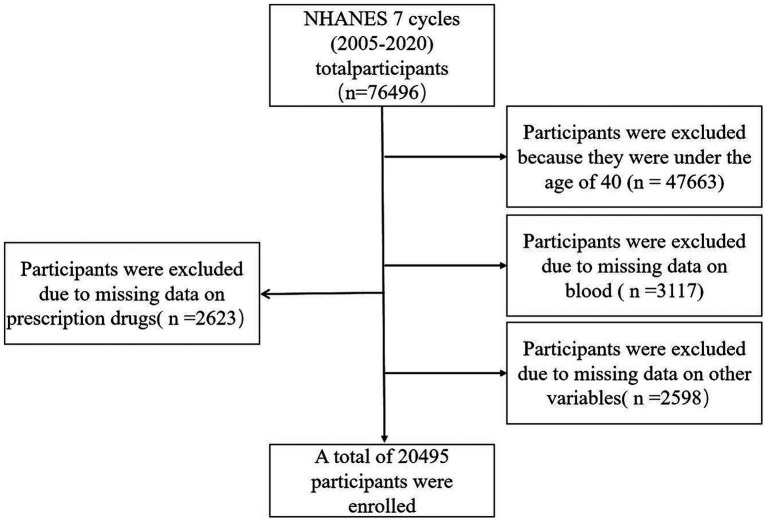
Flow chart of study inclusion. Flowchart of the participants’ selection from NHANES 2005–2020.

Data were obtained from the NHANSE database,^1^ spanning 7 cycles of data from 2005 to 2020. Survey participants underwent blood tests and completed questionnaires. Biochemical test results were screened for participants aged 40 years and older (Zeng et al., 2023a). Basic sociodemographic data and medical history were collected through a family interview. The complete data censoring process is illustrated in Figure 1.

### 2.2 Assessment of PD

The diagnosis of Parkinson’s disease was determined based on whether the participant was taking one or more medications for Parkinson’s disease (DeMarco et al., 2021; Xu et al., 2023; Zeng et al., 2023c). This determination was made by examining responses to questions about prescription drugs. Due to limitations in the medications and codes in NHANES, an individual had to be actively receiving treatment for Parkinson’s disease to be classified as having the condition. Participants who did not report taking anti-Parkinson’s disease medications were considered not to have Parkinson’s disease.

### 2.3 Measurement of serum potassium

Serum specimens are processed, stored, and shipped to the Collaborative Laboratory Services for analysis. Serum calcium, iron, phosphorus, chloride, sodium, and potassium concentration is measured by electrolyte activity in solution. The DxC800 system uses indirect (or diluted) I.S.E. (ion selective electrode) methodology to measure ions in biological fluids. The ferrous ion is immediately complexed with the FerroZine Iron Reagent. The system monitors the change in absorbance at 560 nm at a fixed-time interval. Inorganic phosphorus reacts with ammonium molybdate in an acidic solution to form a colored phosphomolybdate complex. The system monitors the change in absorbance at 365 nm at a fixed-time interval. Calcium, potassium, and potash were calculated from their concentrations by means of the Nernst equation. The NHANES Laboratory provides detailed instructions on collecting and processing specimens.

### 2.4 Measurements of other covariates

The main covariates in the study included demographic characteristics and chronic comorbidities. Respondents provided information on demographic factors such as age, Sex, race, education, marital status, BMI, and smoking status. Chronic comorbidities such as diabetes mellitus, coronary heart disease, hypertension, stroke, and hyperlipidemia were diagnosed by physicians or through self-report questionnaires. With respect to marital status, we categorized married and partnered as married, and divorced and separated, widowed, and never married as unmarried. A trained health technician measured weight and height to calculate BMI, using the formula weight (kg) divided by the square of height (m^2).

### 2.5 Statistical analyses

SPSS was utilized for data extraction and cleaning, while the NHANES R software package (version 4.2.1) and Free Statistics software (version 1.9) were employed for data analysis (Zeng et al., 2023c). Categorical variables were presented as frequencies and percentages, and continuous variables as means and standard deviations (SD). Disparities in continuous and categorical variables were assessed using independent and chi-square tests, respectively. One-way logistic analysis and multivariable logistic regression modeling were conducted to establish the independent association between blood potassium concentration and PD. Four models were developed: Model 1 was unadjusted, Model 2 was adjusted for age, sex, race, education, and marriage, Model 3 was adjusted for age, sex, race, education, marriage, smoking status, and BMI, and Model 4 was adjusted for age, sex, race, education, marriage, smoking status, BMI, and comorbidities such as coronary heart disease, stroke, diabetes, hypertension and hypercholesterolemia. Curve fitting was employed to visualize the potential linear relationship between serum potassium and PD. Subgroup analyses were utilized to explore the impact of age, sex, race, marriage, and smoking status on serum potassium levels in relation to PD. Statistical significance was considered when *p* < 0.05.

## 3 Results

### 3.1 Study population characteristics

A database of 7 cycles from 2005–2020 in NHANES was utilized for this study, encompassing 76,496 potential participants. Participants younger than 40 years (*n* = 47,663) were excluded, along with 3,117 patients with deficiencies in serum calcium, iron, phosphorus, chloride, sodium, and potassium data. Additionally, 2,598 patients with deficiencies in other variables and 2,623 individuals taking prescription medications with deficiencies were excluded. This resulted in a total of 20,495 participants being included, with 403 patients diagnosed with PD and 20,092 non-PD individuals. Specific data can be found in Figure 1, while Table 1 presents the demographic, socioeconomic, co-morbid, and baseline characteristics of the included population. Statistically significant differences between the PD and non-PD groups were observed in age, sex, race, marital, BMI, smoking, presence of coronary heart disease, history of stroke, hypertension, and serum potassium levels.

**Table 1:** The characteristics of participants.

Variables	Non-PD (*n* = 20,092)	PD (*n* = 403)	*p*_value
Age, mean ± SD	60.7 ± 12.1	64.0 ± 13.1	<0.001^**^
Sex, *n* (%)			0.008^**^
Male	9,661 (48.1)	167 (41.4)	
Female	10,431 (51.9)	236 (58.6)	
Race, *n* (%)			<0.001^**^
Non-hispanic white	9,367 (46.6)	262 (65)	
Non-hispanic black	4,401 (21.9)	53 (13.2)	
Mexican American	2,441 (12.1)	30 (7.4)	
Other race	3,883 (19.3)	58 (14.4)	
Marital, *n* (%)			0.003^**^
Married	7,591 (37.8)	181 (44.9)	
Unmarried	12,501 (62.2)	222 (55.1)	
Education, *n* (%)			0.36
Lower high school	4,926 (24.5)	111 (27.5)	
high school	4,652 (23.2)	92 (22.8)	
Over high school	10,514 (52.3)	200 (49.6)	
BMI, mean ± SD	29.8 ± 6.9	30.4 ± 6.7	0.090
Smoking, *n* (%)			0.012^*^
No	10,488 (52.2)	185 (45.9)	
Yes	9,604 (47.8)	218 (54.1)	
Coronary, *n* (%)			0.02^*^
No	18,698 (93.1)	363 (90.1)	
Yes	1,394 (6.9)	40 (9.9)	
Stroke, *n* (%)			<0.001^**^
No	18,891 (94)	350 (86.8)	
Yes	1,201 (6)	53 (13.2)	
Diabetes, *n* (%)			0.095
No	15,340 (76.3)	290 (72)	
Yes	4,103 (20.4)	100 (24.8)	
Pre-diabetes	649 (3.2)	13 (3.2)	
Hypertension, *n* (%)			0.002^**^
No	9,356 (46.6)	157 (39)	
Yes	10,736 (53.4)	246 (61)	
Hypercholesterolemia, *n* (%)			0.198
No	9,924 (49.4)	186 (46.2)	
Yes	10,168 (50.6)	217 (53.8)	
Calcium(mmol/L), Mean ± SD	2.4 ± 0.1	2.3 ± 0.1	0.121
Iron(umol/L), Mean ± SD	14.9 ± 6.0	14.5 ± 5.6	0.127
Phosphorus(mmol/L), Mean ± SD	1.2 ± 0.2	1.2 ± 0.2	0.101
Chloride(mmol/L), Mean ± SD	103.2 ± 3.3	102.9 ± 3.7	0.475
Sodium(mmol/L), Mean ± SD	139.5 ± 2.6	139.6 ± 3.0	0.702
Potassium(mmol/L), Mean ± SD	4.0 ± 0.4	4.1 ± 0.4	<0.001^**^

BMI, body mass index. *p < 0.05, **p < 0.01. *p* < 0.05 was considered statistically significant.

### 3.2 Factors associated with PD

In the overall study population of individuals with PD, univariate regression analysis results indicated a positive association between PD and factors such as age, female, marital, BMI, smoking, coronary, stroke, diabetes, hypertension, and serum potassium (*p* < 0.05) (Table 2). Specifically, age showed a positive correlation with PD occurrence, with an odds ratio (OR) of 1.02 (95% confidence interval: 1.01 ~ 1.03). Females had a higher risk of developing PD compared to males, with an OR of 1.31 (1.07 ~ 1.6) (Hao et al., 2023; Zeng et al., 2023b). Non-Hispanic Black, Mexican American, and Other races exhibited a relatively lower risk of developing PD compared to Non-Hispanic White individuals. Moreover, married individuals had a lower prevalence of PD (OR: 0.74, 95% CI: 0.61 ~ 0.91) compared to unmarried individuals. In terms of educational levels, the prevalence of PD was lower among those with a high school education or above, with OR of 0.88 (0.66 ~ 1.16) and 0.84 (0.67 ~ 1.07) when compared to those with less than a high school education. Patients in the stroke group had a significantly higher risk of PD compared to those without stroke, with an OR and 95% CI of 2.38 (1.77 ~ 3.2). Patients with diabetes mellitus were also more prone to PD compared to non-diabetic individuals, with an OR of 1.29 (1.02 ~ 1.62), while pre-diabetic patients had a smaller association with PD, 1.06 (0.61 ~ 1.85). Additionally, hypertension and coronary heart disease were identified as risk factors for PD, with OR and 95% CI of 1.36 (1.11 ~ 1.67) and 1.48 (1.06 ~ 2.06), respectively. Our study found a positive but not statistically significant correlation between serum sodium levels and the development of PD, 1.01 (0.97 ~ 1.05), contradicting previous research (Mao et al., 2017; Rajput et al., 2021), this may be related to the inconsistency of our sample selection. There may be a negative correlation between serum calcium, iron, and chloride levels and PD, although the relationship with serum iron and chloride levels did not reach statistical significance in our study (Madenci et al., 2012). A positive and statistically significant correlation was observed between serum potassium levels and PD, with an OR of 2.2 (1.73 ~ 2.79) (Table 2).

**Table 2:** Univariate logistics regression analysis.

Variable	OR_(95% CI)	*p*_value
Age	1.02 (1.01 ~ 1.03)	<0.001^**^
Sex		
Male	Ref	Ref
Female	1.31 (1.07 ~ 1.6)	0.008^**^
Race		
Non-hispanic white	Ref	Ref
Non-hispanic black	0.43 (0.32 ~ 0.58)	<0.001^**^
Mexican American	0.44 (0.3 ~ 0.64)	<0.001^**^
Other race	0.53 (0.4 ~ 0.71)	<0.001^**^
Marital		
Unmarried	Ref	Ref
Married	0.74 (0.61 ~ 0.91)	0.004^**^
Education		
Lower high school	Ref	Ref
high school	0.88 (0.66 ~ 1.16)	0.355
Over high school	0.84 (0.67 ~ 1.07)	0.157
BMI	1.01 (1 ~ 1.03)	0.092
Smoking		
No	Ref	Ref
Yes	1.29 (1.06 ~ 1.57)	0.012^*^
Coronary		
No	Ref	Ref
Yes	1.48 (1.06 ~ 2.06)	0.02^*^
Stroke		
No	Ref	Ref
Yes	2.38 (1.77 ~ 3.2)	<0.001^**^
Diabetes		
No	Ref	Ref
Yes	1.29 (1.02 ~ 1.62)	0.03^*^
Pre-diabetes	1.06 (0.6 ~ 1.85)	0.843
Hypertension		
No	Ref	Ref
Yes	1.37 (1.12 ~ 1.67)	0.003^**^
Hypercholesterolemia		
No	Ref	Ref
Yes	1.14 (0.93 ~ 1.39)	0.198
Calcium	0.44 (0.16 ~ 1.24)	0.121
Iron	0.99 (0.97 ~ 1)	0.126
Phosphorus	1.22 (0.71 ~ 2.08)	0.475
Chloride	0.98 (0.95 ~ 1)	0.101
Sodium	1.01 (0.97 ~ 1.05)	0.707
Potassium	2.2 (1.73 ~ 2.79)	<0.001**

OR, odds ratio; CI, confidence interval. *p < 0.05, **p < 0.01. *p* < 0.05 was considered statistically significant.

### 3.3 Multivariable logistics regression analysis of the association between serum potassium and PD

Table 3 presents the OR and 95% CI for the relationship between serum potassium levels and PD following multifactorial logistic regression analysis. In the unadjusted model, a significant correlation of 2.2 (1.74 to 2.8) was observed between serum potassium and PD. Specifically, for each 1-unit increase in serum potassium, there was a 120% increase in the risk of PD. In the multivariate regression model, after adjusting for various factors such as age, sex, race, education, marital, BMI, smoking, coronary, stroke, diabetes, hypertension, and hypercholesterolemia, the OR ranged from 2.2 to 1.86, indicating a strong correlation between serum potassium levels and PD with consistent results (*p* < 0.001). Sensitivity analysis was performed with serum potassium quartiles, and the ORs for Q1, Q2, Q3, and Q4 in model 4 were 1.00, 1.14(0.8 ~ 1.63),1.44(1.06 ~ 1.97), and 1.88(1.38 ~ 2.58), respectively, compared to Quartile 1, participants in Quartile 4 had an association with 88% increased risk of PD (P for trend <0.05).

**Table 3:** Multivariate logistics regression analysis.

	Model 1	Model 2	Model 3	Model 4
	OR(95% CI)	OR(95% CI)	OR(95% CI)	OR(95% CI)
Potassium	2.2(1.74 ~ 2.8)^**^	1.95(1.52 ~ 2.5)^**^	1.9(1.48 ~ 2.45)^**^	1.86(1.45 ~ 2.39)^**^
Quartiles				
Quartiles1	Ref	Ref	Ref	Ref
Quartiles2	1.15(0.81 ~ 1.63)	1.12(0.79 ~ 1.6)	1.12(0.79 ~ 1.59)	1.14(0.8 ~ 1.63)
Quartiles3	1.52(1.12 ~ 2.07)^**^	1.44(1.05 ~ 1.96)^*^	1.42(1.04 ~ 1.93)^*^	1.44(1.06 ~ 1.97)^*^
Quartiles4	2.2(1.2 ~ 1.45)^**^	1.93(1.42 ~ 2.64)^**^	1.88(1.38 ~ 2.57)^**^	1.88(1.38 ~ 2.58)^**^
P for trend	<0.001^**^	<0.001^**^	<0.001^**^	<0.001^**^

Model 1, unadjusted; Model 2, adjusted by age, sex, race, education, marital status; Model 3, adjusted by age, sex, race, education, marital status, BMI, Smoking; Model 4, adjusted by age, sex, race, education, marital status, BMI, smoking, coronary, stroke, diabetes, hypertension, hypercholesterolemia. ^*^p < 0.05, ^**^p < 0.01. *p* < 0.05 was considered statistically significant.

### 3.4 Linear relationship between serum potassium and the risk of PD

**Figure 2:**
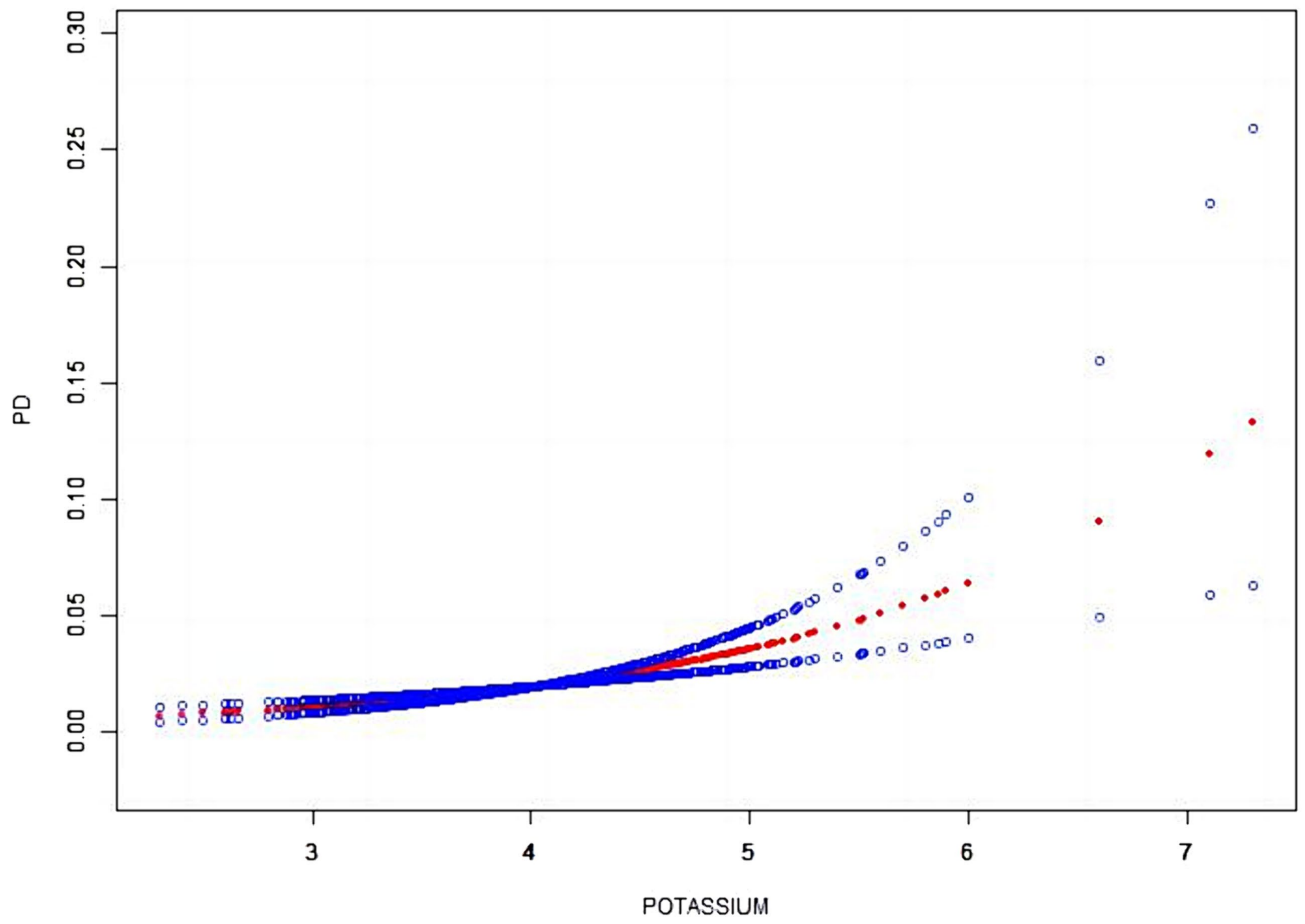
The smooth curve fitting between serum potassium and PD. The x-axis represents serum potassium. While the y-axis represents the 95% confidence interval from the fit. The dashed line indicates an OR of 1, which represents no association between serum potassium and PD risk. The model adjusted by age, sex, race, education, marital, BMI, smoking, coronary, stroke, diabetes, hypertension, hypercholesterolemia.

Our results showed a linear relationship between serum potassium and the risk of developing PD (Figure 2).

### 3.5 Subgroup analyses of factors influencing the association between serum potassium and the presence of PD

**Figure 3:**
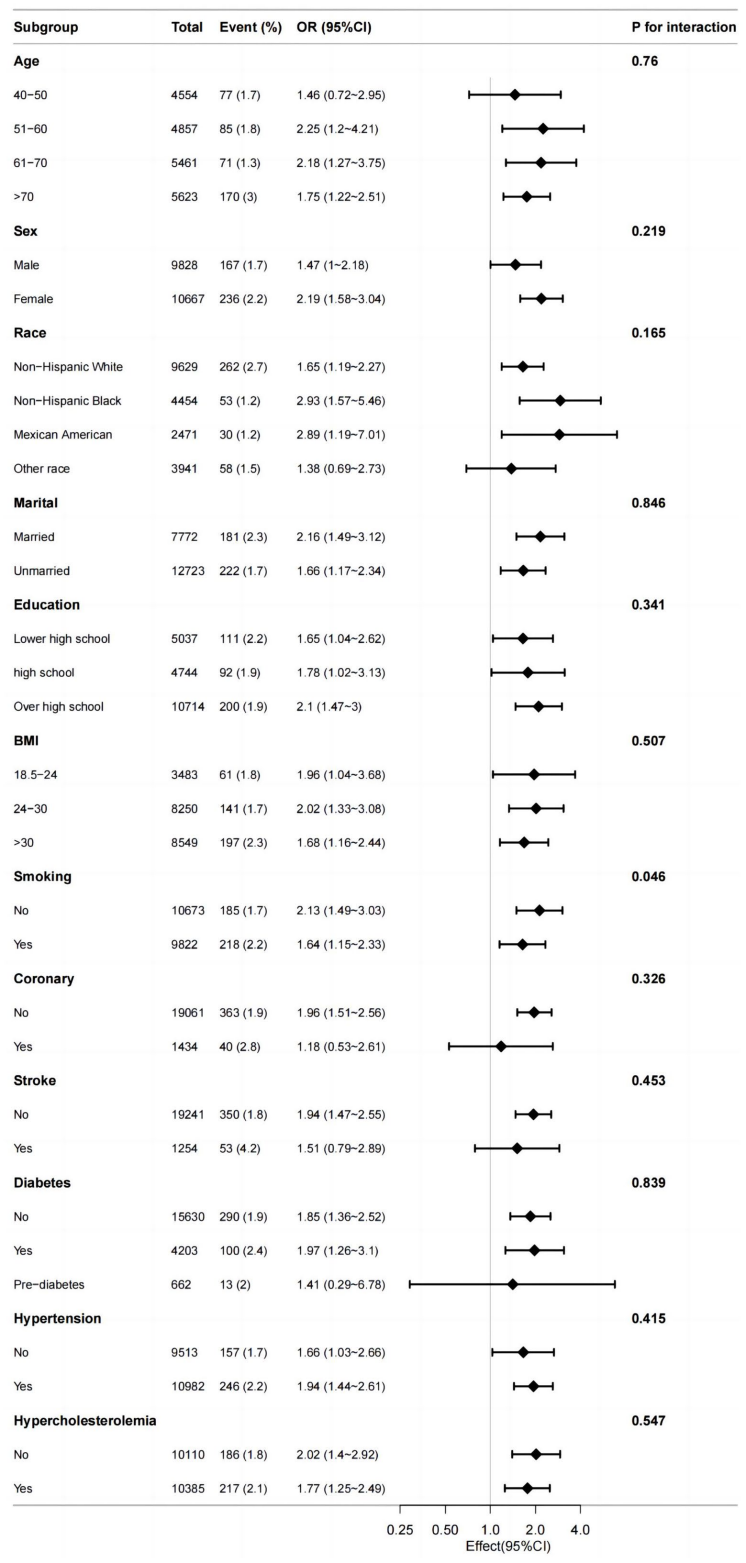
Efect size of serum potassium on the presence of PD in the age, sex, race, marital, BMI, smoking, coronary, stroke, diabetes, hypertension, hypercholesterolemia subgroup.

Stratified analyses were conducted to assess if the association between serum potassium and PD differed based on various demographic and health factors including age, sex, race, marital status, BMI, smoking, coronary, stroke, diabetes, hypertension and hypercholesterolemia. Upon adjusting for covariates, the results indicated that there was no significant interaction between serum potassium and age (*p* = 0.76), sex (*p* = 0.219), race (*p* = 0.165), marital status (*p* = 0.846), education (*p* = 0.341); BMI (*p* = 0.507), coronary (*p* = 0.326), stroke (*p* = 0.453), diabetes (*p* = 0.839), hypertension (*p* = 0.415) or hypercholesterolemia (*p* = 0.547). However, a weak interaction was observed with smoking (*p* = 0.046) (Figure 3).

## 4 Discussion

Potassium, a metallic element essential for the normal functioning of the nervous system, has an unclear role in PD. Exploring the correlation between potassium levels and PD from clinical and social perspectives is crucial. Our study examined the relationship between serum potassium concentration and PD, revealing an increase in serum potassium levels with the prevalence of PD. Multivariable logistic regression, after adjusting for confounders, demonstrated a positive association between elevated serum potassium concentration and the risk of developing PD. Smoothed curve fitting analysis indicated a linear correlation between serum potassium concentration and the risk of PD. In addition to analyzing serum potassium levels, we also examined other serum ion levels in the study. The results indicated a decrease in serum calcium, iron, and chloride levels in the PD population, serum sodium, phosphorus levels showed a slight increase in the PD population, these changes were not deemed statistically significant.

Previous studies have yielded conflicting results regarding potassium concentrations in patients with PD. A study involving 120 PD patients found a non-statistically significant reduction in potassium in scalp hair and blood compared to healthy controls (Rajput et al., 2021). Our study utilized data from the NHANES, benefiting from a large sample size of nationally representative adults in the US, thereby increasing reliability and precision. Contrary to previous findings, our study revealed a higher serum potassium concentration (4.1 ± 0.4 mmol/L) in PD patients compared to non-PD patients, indicating a positive correlation between serum potassium levels and PD. Multifactorial regression analysis, with adjustments for confounding variables, further supported the association between serum potassium levels and PD prevalence.

Potassium, a major intracellular cation, plays a crucial role in various bodily functions. It is essential for maintaining normal neuromuscular function, regulating blood pH, and preserving cell fluid levels. Additionally, potassium is involved in establishing cellular resting membrane potential, regulating neurotransmitter release, and maintaining cell volume and homeostasis (Chen et al., 2018). In brain tissue, extracellular potassium levels are lower than in the rest of the body, contributing to stable brain function. High potassium levels have been linked to increased neuronal excitability (Somjen, 2002), leading to depolarization of neuronal membranes and inhibition of synaptic transmission. These findings highlight the metabolic and regulatory significance of potassium levels in the central nervous system, with potential implications for neurodegenerative diseases (Ha et al., 2016). Under normal conditions, brain ion levels are independently regulated from plasma levels through active transport mechanisms involving the choroid plexus epithelium, cerebral capillary endothelium, and astrocytes (Schain, 1964; Somjen, 2002).Neurodegeneration impacts the function of blood vessels and the blood–brain barrier (Somjen, 2002). A study examining the relationship between serum potassium and Lewy body dementia revealed that lower potassium levels at disease onset were associated with reduced amyloid presence in cerebrospinal fluid as individuals aged. Autopsy findings indicated that serum potassium levels were more closely linked to cognitive performance when there was more α-syn pathology in the brain. Moreover, high potassium intake in early life may heighten the risk of mild cognitive impairment later on (Giil et al., 2019). Furthermore, research has demonstrated that elevated potassium levels post-trauma can lead to cerebrovascular cell swelling or altered vascular reactivity (Reinert et al., 2000). When it comes to AD, cerebrospinal fluid potassium levels were within normal range in AD patients, but serum potassium levels may show a slight elevation (Roberts et al., 2016).

Elevated blood potassium levels in PD may be linked to excessive potassium intake. Orthostatic hypotension is a common symptom in individuals with PD, leading to increased cognitive dysfunction and dyskinesia (McDonald et al., 2016; Dommershuijsen et al., 2021). To prevent these issues, patients with PD may be advised to increase their fluid and electrolyte intake, potentially resulting in elevated blood potassium levels. Moreover, a high-potassium diet could exacerbate dyskinesia symptoms in PD patients (Alizadeh et al., 2023). Furthermore, damage to potassium channels in the substantia nigra and striata may also play a role in the elevated potassium levels. These channels, which are selective pores for potassium ions in the cell membrane, are widely distributed and regulate various biological functions. Potassium channels play a crucial role in regulating the flow of potassium ions in and out of cells, impacting cellular potential, excitability, and overall function. Dysregulation of potassium channel function can disrupt the balance of potassium levels inside and outside cells, leading to abnormal serum potassium concentrations that can affect neuromuscular function. Research indicates that various potassium channels are expressed in the substantia nigra-striatal system, influencing neurotransmitter release, neuronal excitability, and cell volume (Zhang et al., 2020). Specifically, Kv channels are a type of transmembrane channels that facilitate the release of potassium ions and respond to changes in cell membrane potential. In nigral dopaminergic neurons, Kv1.3, Kv2.1, Kv3.2, and Kv3.3 channels can be identified. Among these, Kv1.3 is composed of four subunits, each containing six transmembrane segments and a voltage transducer that opens the channel to allow potassium efflux upon detecting membrane depolarization (Sarkar et al., 2020). Studies have observed increased expression of Kv1.3 and potassium efflux in postmortem PD patients, as well as in animal models induced by MPTP, α-syn transfection, and MitoPark. Furthermore, Kv1.3 has been found to be upregulated in microglia, potentially influenced by α-syn stimulation, leading to enhanced potassium efflux (Pike et al., 2022).

Our study has several advantages and implications. It is worth noting that the analysis performed a smoothed curve fitting analysis to indicate the linear relationship between serum potassium and PD. In addition, a stratified subgroup analysis was conducted to further investigate the relationship between serum potassium and PD across different population groups, which suggests that we need to implement more precise prevention strategies for PD. The study has several limitations that need to be addressed. Firstly, the lack of measurements related to renal function and potassium intake in our investigation limits our understanding of potassium homeostasis. Secondly, the cross-sectional nature of the study using NHANES data prevents us from establishing causality, emphasizing the need for future prospective cohort studies to validate our results. Thirdly, the identification of PD patients based solely on medication intake may introduce bias, as individuals with a history of stroke or psychiatric disorders may also be taking similar medications. Despite these limitations, conducting a multicenter controlled trial to confirm our findings is essential.

## 5 Conclusion

The level of serum potassium in patients with PD is associated with a higher risk of prevalence. Therefore, low serum potassium levels may have a protective effect against PD.

## Data availability statement

Publicly available datasets were analyzed in this study. This data can be found here: https://www.cdc.gov/nchs/nhanes/index.htm.

## Ethics statement

The studies involving humans were approved by NCHS Ethics Review Board; National Center for Health Statistics; Centers for Disease Control and Prevention. The studies were conducted in accordance with the local legislation and institutional requirements. The participants provided their written informed consent to participate in this study.

## Author contributions

XZ: Writing – original draft. JZ: Writing – original draft. YL: Data curation, Writing – original draft. XS: Data curation, Writing – original draft. XL: Methodology, Writing – original draft. JR: Methodology, Writing – original draft. QL: Software, Writing – original draft. DH: Software, Writing – original draft. TP: Formal analysis, Writing – original draft. YS: Formal analysis, Writing – original draft. DW: Writing – review & editing. XC: Writing – review & editing.

## Footnotes

^1^ https://www.cdc.gov/nchs/nhanes/index.htm

## References

[ref1] Alizadeh M. Kheirouri S. Keramati M. (2023). What dietary vitamins and minerals might be protective against Parkinson’s disease? Brain Sci. 13:1119. doi: 10.3390/brainsci13071119, PMID: 37509049 PMC10377174

[ref2] Camponeschi F. Valensin D. Tessari I. Bubacco L. Dell’Acqua S. Casella L.. (2013). Copper(I)-α-Synuclein interaction: structural description of two independent and competing metal binding sites. Inorg. Chem. 52, 1358–1367. doi: 10.1021/ic302050m, PMID: 23343468

[ref3] Chen X. Xue B. Wang J. Liu H. Shi L. Xie J. (2018). Potassium channels: a potential therapeutic target for Parkinson’s disease. Neurosci. Bull. 34, 341–348. doi: 10.1007/s12264-017-0177-3, PMID: 28884460 PMC5856711

[ref4] Davies P. Wang X. Sarell C. J. Drewett A. Marken F. Viles J. H.. (2011). The Synucleins are a family of redox-active copper binding proteins. Biochemistry 50, 37–47. doi: 10.1021/bi101582p, PMID: 21117662

[ref5] DeMarco E. C. Al-Hammadi N. Hinyard L. (2021). Exploring treatment for depression in Parkinson’s patients: a cross-sectional analysis. Int. J. Environ. Res. Public Health 18:8596. doi: 10.3390/ijerph18168596, PMID: 34444343 PMC8392211

[ref6] Dommershuijsen L. J. Heshmatollah A. Mattace Raso F. U. S. Koudstaal P. J. Ikram M. A. Ikram M. K. (2021). Orthostatic hypotension: a prodromal marker of Parkinson’s disease? Mov. Disord. 36, 164–170. doi: 10.1002/mds.28303, PMID: 32965064 PMC7891584

[ref7] Follett J. Darlow B. Wong M. B. Goodwin J. Pountney D. L. (2013). Potassium depolarization and raised calcium induces α-synuclein aggregates. Neurotox. Res. 23, 378–392. doi: 10.1007/s12640-012-9366-z, PMID: 23250862

[ref8] Fraga C. G. (2005). Relevance, essentiality and toxicity of trace elements in human health. Mol. Asp. Med. 26, 235–244. doi: 10.1016/j.mam.2005.07.013, PMID: 16125765

[ref9] Giil L. M. Solvang S.-E. H. Giil M. M. Hellton K. H. Skogseth R. E. Vik-Mo A. O.. (2019). Serum potassium is associated with cognitive decline in patients with Lewy body dementia. JAD 68, 239–253. doi: 10.3233/JAD-181131, PMID: 30775974

[ref10] Ha Y. Jeong J. A. Kim Y. Churchill D. G. (2016). Sodium and potassium relating to Parkinson’s disease and traumatic brain injury. Met. Ions Life Sci. 16, 585–601. doi: 10.1007/978-3-319-21756-7_16, PMID: 26860312

[ref11] Hao X. Li H. Li Q. Gao D. Wang X. Wu C.. (2023). Dietary vitamin E intake and risk of Parkinson’s disease: a cross-sectional study. Front. Nutr. 10:1289238. doi: 10.3389/fnut.2023.1289238, PMID: 38249609 PMC10799344

[ref12] Jankovic J. Tan E. K. (2020). Parkinson’s disease: etiopathogenesis and treatment. J. Neurol. Neurosurg. Psychiatry 91, 795–808. doi: 10.1136/jnnp-2019-322338 32576618

[ref13] Lothian A. Hare D. J. Grimm R. Ryan T. M. Masters C. L. Roberts B. R. (2013). Metalloproteomics: principles, challenges and applications to neurodegeneration. Front. Aging Neurosci. 5:35. doi: 10.3389/fnagi.2013.00035, PMID: 23882215 PMC3714543

[ref14] Madenci G. Bilen S. Arli B. Saka M. Ak F. (2012). Serum iron, vitamin B12 and folic acid levels in Parkinson’s disease. Neurochem. Res. 37, 1436–1441. doi: 10.1007/s11064-012-0729-x, PMID: 22367474

[ref15] Mao C. Zhong C. Yang Y. Yang Y. Wang F. Chen J.. (2017). Serum sodium and chloride are inversely associated with dyskinesia in Parkinson’s disease patients. Brain Behav. 7:e00867. doi: 10.1002/brb3.867, PMID: 29299386 PMC5745246

[ref16] McDonald C. Newton J. L. Burn D. J. (2016). Orthostatic hypotension and cognitive impairment in Parkinson’s disease: causation or association? Mov. Disord. 31, 937–946. doi: 10.1002/mds.26632 27091624

[ref17] Moons R. Konijnenberg A. Mensch C. Van Elzen R. Johannessen C. Maudsley S.. (2020). Metal ions shape α-synuclein. Sci. Rep. 10:16293. doi: 10.1038/s41598-020-73207-9, PMID: 33004902 PMC7529799

[ref18] Philbert S. A. Xu J. Scholefield M. Church S. J. Unwin R. D. Cooper G. J. S. (2022). Contrasting sodium and potassium perturbations in the Hippocampus indicate potential Na+/K+-ATPase dysfunction in vascular dementia. Front. Aging Neurosci. 14:822787. doi: 10.3389/fnagi.2022.822787, PMID: 35153731 PMC8832097

[ref19] Pike A. F. Szabò I. Veerhuis R. Bubacco L. (2022). The potential convergence of NLRP3 inflammasome, potassium, and dopamine mechanisms in Parkinson’s disease. NPJ Parkinson’s Dis. 8:32. doi: 10.1038/s41531-022-00293-z, PMID: 35332154 PMC8948240

[ref20] Rajput K. Afridi H. I. Kazi T. G. Talpur F. N. Baig J. A. (2021). Sodium, potassium, calcium, and magnesium in the scalp hair and blood samples related to the clinical stages of the Parkinson’s disease. Biol. Trace Elem. Res. 199, 2582–2589. doi: 10.1007/s12011-020-02399-y, PMID: 32959340

[ref21] Reinert M. Khaldi A. Zauner A. Doppenberg E. Choi S. Bullock R. (2000). High level of extracellular potassium and its correlates after severe head injury: relationship to high intracranial pressure. J. Neurosurg. 93, 800–807. doi: 10.3171/jns.2000.93.5.0800, PMID: 11059661

[ref22] Riggs J. E. (1989). Neurologic manifestations of fluid and electrolyte disturbances. Neurol. Clin. 7, 509–523. doi: 10.1016/S0733-8619(18)30797-7 2671634

[ref23] Roberts B. R. Doecke J. D. Rembach A. Yévenes L. F. Fowler C. J. McLean C. A.. (2016). Rubidium and potassium levels are altered in Alzheimer’s disease brain and blood but not in cerebrospinal fluid. Acta Neuropathol. Commun. 4:119. doi: 10.1186/s40478-016-0390-8, PMID: 27842602 PMC5109650

[ref24] Roberts B. R. Ryan T. M. Bush A. I. Masters C. L. Duce J. A. (2012). The role of metallobiology and amyloid-β peptides in Alzheimer’s disease. J. Neurochem. 120, 149–166. doi: 10.1111/j.1471-4159.2011.07500.x, PMID: 22121980

[ref25] Sarkar S. Nguyen H. M. Malovic E. Luo J. Langley M. Palanisamy B. N.. (2020). Kv1.3 modulates neuroinflammation and neurodegeneration in Parkinson’s disease. J. Clin. Invest. 130, 4195–4212. doi: 10.1172/JCI136174, PMID: 32597830 PMC7410064

[ref26] Schain R. J. (1964). Cerebrospinal fluid and serum cation levels. Arch. Neurol. 11, 330–333. doi: 10.1001/archneur.1964.00460210108012 14170639

[ref27] Somjen G. G. (2002). Ion regulation in the brain: implications for pathophysiology. Neuroscientist 8, 254–267. doi: 10.1177/1073858402008003011 12061505

[ref28] Sridharan M. R. Flowers N. C. (1986). Hyperkalemia and Wolff-Parkinson-white type preexcitation syndrome. J. Electrocardiol. 19, 183–187. doi: 10.1016/s0022-0736(86)80026-7, PMID: 3711755

[ref29] Waldron K. J. Rutherford J. C. Ford D. Robinson N. J. (2009). Metalloproteins and metal sensing. Nature 460, 823–830. doi: 10.1038/nature08300 19675642

[ref30] Xu S. Li W. Di Q. (2023). Association of Dietary Patterns with Parkinson’s disease: a cross-sectional study based on the United States National Health and nutritional examination survey database. Eur. Neurol. 86, 63–72. doi: 10.1159/000527537, PMID: 36470220

[ref31] Zeng Z. Cen Y. Luo X. (2023a). Association between blood selenium with parkinson’s disease in the US (NHANES 2011-2020). Environ. Sci. Pollut. Res. Int. 30, 117349–117359. doi: 10.1007/s11356-023-30337-7, PMID: 37864700

[ref32] Zeng Z. Cen Y. Wang L. Luo X. (2023b). Association between dietary inflammatory index and Parkinson’s disease from National Health and nutrition examination survey (2003-2018): a cross-sectional study. Front. Neurosci. 17:1203979. doi: 10.3389/fnins.2023.1203979, PMID: 37547135 PMC10398569

[ref33] Zeng Z. Cen Y. Xiong L. Hong G. Luo Y. Luo X. (2023c). Dietary copper intake and risk of Parkinson’s disease: a cross-sectional study. Biol. Trace Elem. Res. 202, 955–964. doi: 10.1007/s12011-023-03750-9, PMID: 37462848 PMC10803382

[ref34] Zhang L. Zheng Y. Xie J. Shi L. (2020). Potassium channels and their emerging role in parkinson’s disease. Brain Res. Bull. 160, 1–7. doi: 10.1016/j.brainresbull.2020.04.004, PMID: 32305406

